# Long term management of intracranial epidermoids balancing extent of resection and functional preservation in a 20 year institutional experience

**DOI:** 10.1038/s41598-025-90333-4

**Published:** 2025-02-17

**Authors:** Mazin Omer, Julia M. Nakagawa, Arthur H. A. Sales, Theresa Bettina Loidl, Christian Scheiwe, Jürgen Beck, Jürgen Grauvogel, Christine J. Gizaw

**Affiliations:** Department of Neurosurgery, Medical Center – Faculty of Medicine, University of Freiburg, Freiburg, Germany

**Keywords:** Intracranial epidermoid lesions, CPA epidermoid, Skull base surgery, Neurosurgical removal, Cerebellopontine angle, Functional preservation, Neurology, Oncology

## Abstract

Epidermoid lesions account for 1% of intracranial neoplasms. Surgical management is challenging due to their adhesion to critical neurovascular structures and tendency for recurrence. The current study examines surgical outcomes, extent of resection, and recurrence rates during long-term follow-up. A retrospective analysis was conducted on patients treated for epidermoid lesions between 2000 and 2021, focusing on clinical and radiological outcome and long-term symptom development. Among 55 patients (56.4% male), the majority harbored lesions in the cerebellopontine angle (75.3%). The mean age at surgery was 41.3 years, with an average follow-up of 82 months. Total removal was achieved in 61% of cases, with 75% of them remaining recurrence-free. In comparison, 35% of near-total removal and 25% of subtotal removal remained recurrence-free. Immediate symptom improvement was similar after total and non-total removal (12.6% vs. 10.5%), but long-term improvement was higher after total removal (43% vs. 27%). Transient cranial nerve deficits occurred in 25% of total and in 32% of non-total removal cases, with similar rates of minor complications. Total removal provided better long-term symptom control and lower recurrence rates without significantly increasing neurological deficits, supporting it as the preferred surgical strategy while maintaining functional preservation.

## Introduction

Intracranial epidermoid lesions, alternatively named pearly tumors in existing literature, represent a subset of congenital extra-axial lesions^1^. It is believed that they originate from the entrapment of ectodermal squamous epithelium during the closure of the neural tube, primarily situated lateral to the midline^2^. Constituting a minority of intracranial neoplasms, ranging from 0.2 to 1.8%, epidermoid lesions often evolve asymptomatically due to their slow growth^3,4^. Manifesting a unique behavior, they tend to propagate along physiological cleavage planes, gradually occupying available subarachnoid spaces encompassing fissures, sulci, cisterns, and ventricles^5,6^. As they expand, these lesions adapt to the contours of these cavities. Rather than disrupting vital structures including nearby normal neuronal and vascular elements, epidermoids intricately adhere to and envelop them throughout their growth trajectory.

Approximately 40–50% of epidermoid lesions are located in the “cerebellopontine angle” (CPA), which present a formidable challenge in neurosurgical contexts, 30% are found in the parasellar region, 5–18% in the fourth ventricle and less commonly in the middle cranial fossa^7,8,9,10.11^.Furthermore, not only that they are located near critical neurovascular structures, often enshrouding them, but segments of the lesions’ capsule also frequently become densely attached to these structures, complicating their removal immensely. Accordingly, these lesions have the potential to induce damage in cranial nerves, leading to e.g., facial nerve affection and trigeminal neuralgia. Permanent cranial nerve damage, which can persist even after surgery, arises when the lesion adheres to the nerve, causing ischemia^12^. Despite their benign nature, these lesions carry a significant risk of perioperative complications, encompassing enduring cranial nerve deficits, hydrocephalus, and aseptic meningitis, attributed to breakdown products like keratin and cholesterol originating from the desquamation of epithelial cells^13^.

Nonetheless, the primary objective of any intervention remains complete excision while preserving neurological function^14^. However, the decision to pursue complete removal of epidermoid lesions needs extensive evaluation at times, striking a delicate balance between the benefits of radical resection and the perils of surgical complications or recurrence, all while prioritizing functional preservation.

The integration of endoscopic techniques alongside microsurgery improves efforts to achieve complete removal^14,15^. Given the biological attributes of epidermoid lesions - including the presence of cholesterol crystals and keratin, a pliable consistency, limited vascularity, and an intact capsule - the utilization of endoscopes becomes advantageous in surgical procedures. A neuroendoscopic approach offers superior illumination and enhanced magnification with clear visualization of anatomical features encompassing the lesion, blood vessels, and nerves.

In this study, we report our 20-year institutional experience with intracranial epidermoid lesions, with special emphasis on the correlation between surgical outcome and extent of removal, as well as timing of recurrence or regrowth.

## Methods

Between January 1, 2000, and December 31, 2021, a cohort of 55 consecutive patients diagnosed with intracranial epidermoid lesions underwent surgical interventions at our neurosurgical tertiary care center specialized in skull base surgery. While the cohort consists of 55 patients, it encompasses a total of 65 operative cases. Here, “cases” includes not only initial surgeries, but also recurrent operations performed on the same patients. The study received ethical approval from the Ethics Committee of the University of Freiburg, Germany (Ethics Vote number: 23-1254-S1-retro, Date: 03.08.2023), in compliance with the Declaration of Helsinki. Due to the retrospective nature of the study, the Ethics Committee waived the need of obtaining informed consent. A comprehensive retrospective analysis of clinical, surgical, and neuroradiologic records was undertaken to extract a spectrum of data, encompassing age, gender, clinical manifestations and symptoms, neuroradiologic findings, surgical approaches and extent of removal, neurological findings and complications, recurrence or regrowth and final clinical outcomes.

**Fig. 1:**
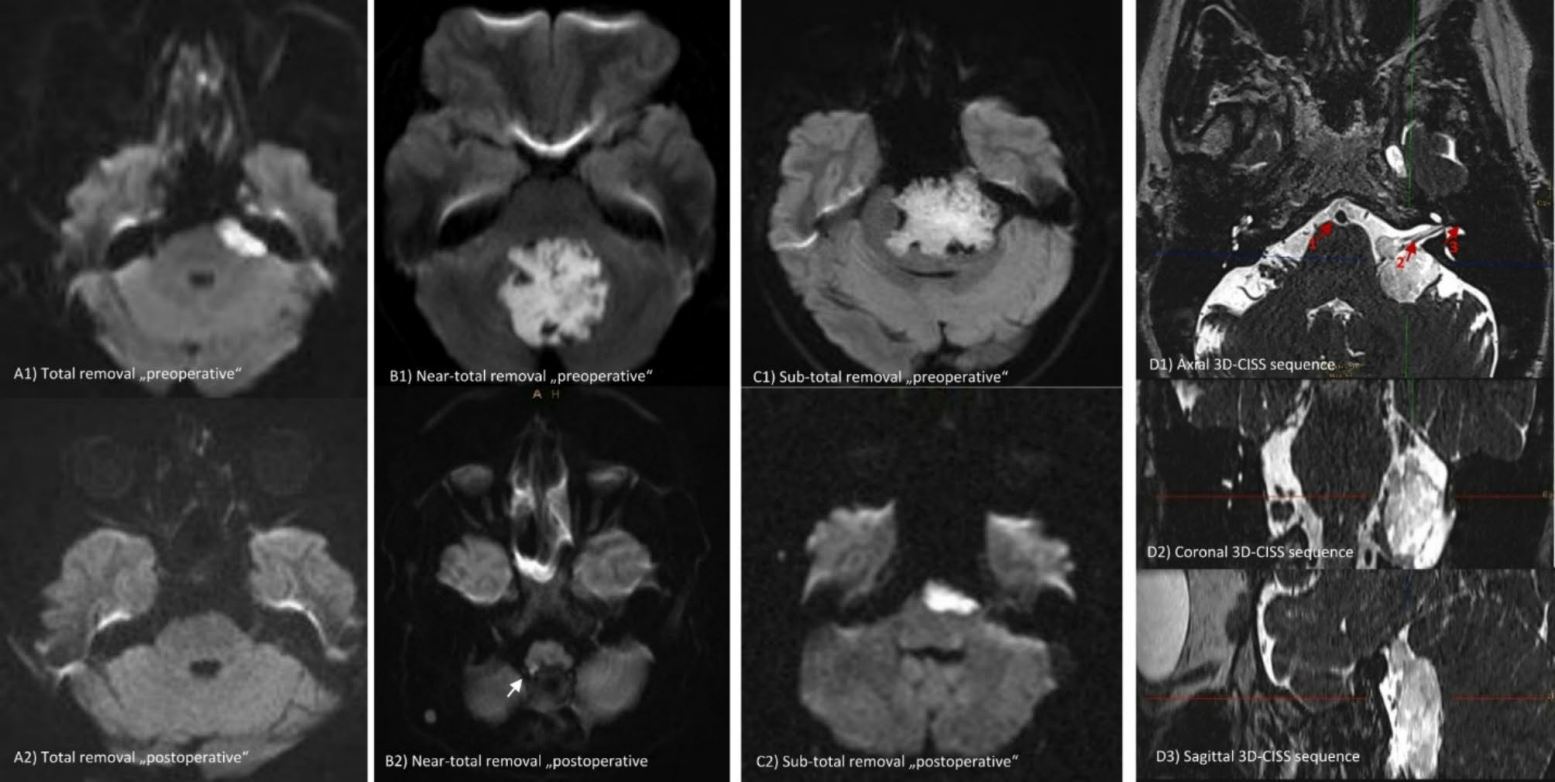
Preoperative and postoperative DWI MRI studies illustrating the classification of the extent of resection: **(A)** Total removal, **(B)** Near-total removal, and **(C)** Subtotal removal. **(D)** 3D-CISS sequence showing neurovascular structures surrounding an epidermoid tumor in the cerebellopontine angle (CPA). Specific structures labeled in **(D1)**:1 – Basilar artery, 2 – Cranial nerves VII and VIII, 3 – Internal acoustic meatus.

The extent of removal was assessed based on first available postoperative MR imaging, with special emphasis on diffusion-weighted imaging to accurately identify residual epidermoid tissue. Here, early DWI scans were consistently utilized as the follow-up imaging modality for all cases, with a median time from surgery to the first MRI in our cohort of 3 months (Interquartile Range “IQR”: 3–4 months). The extent of removal was classified into three categories: “total,” “near-total,” and “subtotal” removal. Specifically, “total removal” was defined as the complete absence of diffusion-restricted remnants on MRI. “Near-total removal” was classified as achieving a resection of ≥ 98–99% of the lesion, while any residual lesion beyond this threshold was considered “subtotal removal” (Fig. 1). Furthermore, the use of endoscope war reported in the result section, the decision to use endoscopy was made at the discretion of the surgeon, based on mainly the intraoperative finding including nonetheless, factors like blind spot visualization, tumor adherence, anatomical constraints and facilitate safer dissection around critical neurovascular structures. Long-term radiological follow-up was conducted, with Kaplan–Meier recurrence-free survival curves generated for the total removal group, and regrowth-free periods assessed for the near-total and subtotal removal groups. Concerning the predictors of lesion regrowth, adhesion severity was determined through a detailed review of operative reports, as well as preoperative and postoperative MRI scans, categorizing lesions as either less adhesive or more adhesive. Additionally, postoperative clinical assessments and one-year follow-up examinations were obtained for each patient to establish longitudinal evaluation of neurological condition. Functional status was appraised using the Modified Rankin Scale (mRS) (preoperatively, at discharge and at subsequent follow-ups).

## Results

### Demographics

Table 1 provides an overview of the demographic characteristics of the patient cohort. The study included 55 patients, of which 45 were undergoing their first operation in life at our clinic and 10 had previously been operated due to their epidermoid lesion at an external facility. 56.4% of the patients were male (31 patients) and 43.6% female (24 patients). The mean age of the participants at surgery in our clinic was 41.32 years. The mean follow-up period was 82.02 months, ranging from 13 to 199 months.

**Table 1:** Demographics of the patients.

Parameter	Value
Total number of patients First operation at study center Previous operation at external facility	55 (100%) 45 (81.8%) 10 (18.2%)
**Sex**	
Male	31 (56.4%)
Female	24 (43.6%)
**Age (years)**, mean ± SD	41.32 ± 16,8
**Follow up period “months”**, Mean (range), ± SD	82.02 (13–199), ± 44.2

### Symptomatology

**Fig. 2:**
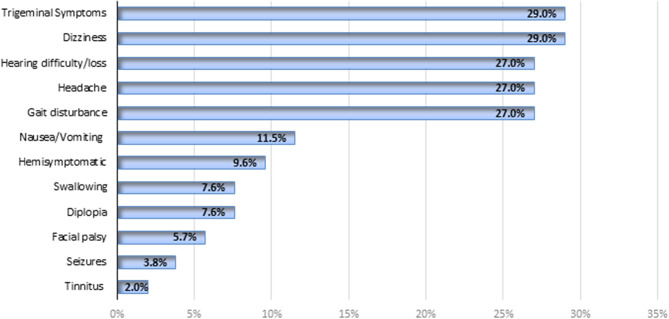
Distribution of preoperative symptomatology.

The main preoperative symptoms experienced by patients with intracranial epidermoid lesions included dizziness and trigeminal symptoms, each affecting 29% of the patients (Fig. 2). This was followed by gait disturbance, headache, and hearing difficulty/loss, each present in 27% of the patients. Other symptoms like nausea/vomiting (11.5%), hemisymptoms (9.6%), swallowing disturbance, and diplopia (each 7.6%) were less common. The least frequent symptoms in our cohort observed were facial palsy (5.7%), seizures (3.8%), and tinnitus (2%).

### Surgical aspects and extent of resection

In Table 2, the data shows that among the 65 cases studied, most lesions were located in the cerebellopontine angle (CPA) and prepontine area, accounting for 49 cases (75.3%), 9 cases (13.9%) were located supratentorially and 7 cases (10.8%) in the fourth ventricle. A retrosigmoid approach was predominantly chosen for lesions located in the cerebellopontine angle and prepontine area, used in 79.6% of cases, and mainly a pterional approach for supratentorial (77.7%) and a median suboccipital approach for all lesions located in the fourth ventricle (100%). In CPA and prepontine location, total removal was achieved in 60.4% of the cases, near-total removal in 27% and subtotal resection in 12.5%. In supratentorial lesions, total resection was achieved in 66.6% of the cases, near-total resection in 22.2% and subtotal resection in 11%. 57.1% of the lesions located in fourth ventricle were totally resected and in 42.8% of the cases near-total resection was achieved. Endoscopic assistance was employed in 22 cases (33.8%) among the cohort. The extent of lesion removal showed comparable outcomes between the endoscopic-assisted and non-endoscopic groups, with total resection achieved in 54.5% and 64.2% of cases, respectively, with similar rates for near-total and subtotal removal. Neurological improvement of preoperative symptoms was slightly higher in the endoscopic assisted group (22.7% vs. 13%). Intraoperative monitoring was used in 56 cases (86%).

**Table 2:** Lesion’s location, surgical approach and extension of removal.

Tumor Location	No. of cases (%)	Surgical Approach*	Total resection	Near total resection	Subtotal resection	Endoscopic assisted	Monitoring
cerebellopontine angle (CPA) and prepontine region	49 (75.3%)	**R**etrosegmoidal **=** 39 (79.6%) **O**ther **S**uboccipital*=6 (12%) **P**resigmoid **=** 1 (2%) **T**emporal **=** 2 (4%) **P**terional **=** 1 (2%)	29 (60.4%)	13 (27%)	6 (12.5%)	18 (36.7%)	44 (89.7%)
Supratentorial	9 (13.9%)	**P**terional **=** 7 (77.7%) **T**emporal **=** 1 (11.1%) **S**ubtemporal **=** 1 (11.1%)	6 (66.6%)	2 (22.2%)	1 (11.1%)	3 (33.3%)	6 (66.6%)
4th Ventricle	7 (10.8%)	**M**edian **S**uboccipital **=** 1 (100%)	4 (57.1%)	3 (42.8%)	0	1 (14.2%)	6 (85.7%)
**Total**	65 (100%)	65 (100%)	39 (61%)	18 (27.6%)	7 (10.7%)	22 (33.8%)	56 (86%)

* Other Suboccipital (LS: Lateral Suboccipital MS: Median Suboccipital, S: Suboccipital).

### Development of neurological status and functional outcome

Table 3 assesses the Postoperative Neurological Status comparing immediate postoperative results and follow-up at one year with the preoperative score in the two groups - total and not-total removal (“not-total” encompasses both near-total and subtotal resections). The analysis revealed that new postoperative deficits occurred in 20 cases within the total resection group compared to 15 cases in the not-total resection group. Importantly, by the one-year follow-up, the incidence of symptom improvement—considering both preoperative symptoms and new postoperative deficits—was notably higher in the total removal group (43%) than in the not-total removal group (28%).

Additionally, the likelihood of symptoms remaining stable at follow-up was greater in the not-total resection group (62%) compared to the total removal group (46.8%). Despite these differences in improvement and stability rates, both groups demonstrated a comparable rate of persistently worse symptoms at follow-up (8.8% for total removal vs. 10% for not-total removal).

**Table 3:** Neurological status, immediate postoperative and at one year follow-up in comparison to preoperative status, comparing total vs. not-total removal groups. CN = cranial nerve.

Neurological deficits	Immediate postoperative status										Follow Up status					
	Total Removal					Not-total removal					Total Removal			Not total removal		
	Total number	Postoperative development of pre-existing preoperative deficits			New deficits after surgery	Total Number	Postoperative development of pre-existing preoperative deficits			New deficits after surgery	Development of deficits (compared to immediate postoperative status)			Development of deficits (compared to immediate postoperative status)		
		worse	better	stable			worse	better	stable		worse	better	stable	worse	better	stable
CN 3/4/6	10	0	0	4 (40%)	6 (60%)	8	0	0	3 (37.5%)	5 (62.5%)	1 (10%)	5 (50%)	4 (40%)	1 (12.5%)	2 (25%)	5 (62.5%)
CN 5	12	1 (8.3%)	1 (8.3%)	10 (83.3%)	0	9	4 (44.4%)	1 (11.1%)	4 (44.4%)	0	3 (25%)	2 (16.6%)	7 (58.3%)	1 (11.2%)	3 (33.3%)	5 (45%)
CN 7	8	1 (12.5%)	0	1 (12.5%)	6 (75%)	5	0	1 (20%)	2 (40%)	2 (40%)	0	5 (62.5%)	3 (37.5%)	0	1 (20%)	4 (80%)
CN 8	15	1 (6.6%)	1 (6.6%)	11 (73.3%)	2 (13.3%)	4	2 (50%)	0	2 (50%)	0	0	6 (40%)	9 (60%)	0	3 (42.8%)	4 (57.2%)
Caudal Cranial nerves 9–12	9	0	2 (22.2%)	3 (33.3%)	4 (44.4%)	6	0	1 (16.6%)	1 (16.6%)	4 (66.6%)	1 (11.2%)	3 (33.3%)	5 (55.5%)	0	3 (50%)	3 (50%)
Cerebellar signs (Ataxia and dizziness)	17	1 (5.8%)	4 (23.5%)	11 (64.7%)	1 (5.8%)	9	0	1 (11.1%)	7 (77.7%)	1 (11.1%)	2 (11.7%)	9 (53%)	6 (35.2%)	1 (11.1%)	1 (11.1%)	7 (77.7%)
Seizures	1	1	0	0	0	1	0	1 (100%)	0	0	0	1 (100%)	0	1 (100%)	0	0
Hemi-symptomatic	7	1 (14.2%)	2 (28.5%)	3 (42.8%)	1 (14.2%)	5	1 (20%)	0	1 (20%)	3 (60%)	1 (14%)	3 (42.5%)	3 (42.8%)	1 (20%)	1 (20%)	3 (60%)
**Total**	79	6 (7.5%)	10 (12.6%)	43 (54.4%)	20 (25.3%)	47	7 (15%)	5 (10.5%)	20 (42.5%)	15 (32%)	11 (8.8%)	33 (43.0%)	35 (46.8%)	5 (10%)	14 (28%)	31 (62%)

**Fig. 3:**
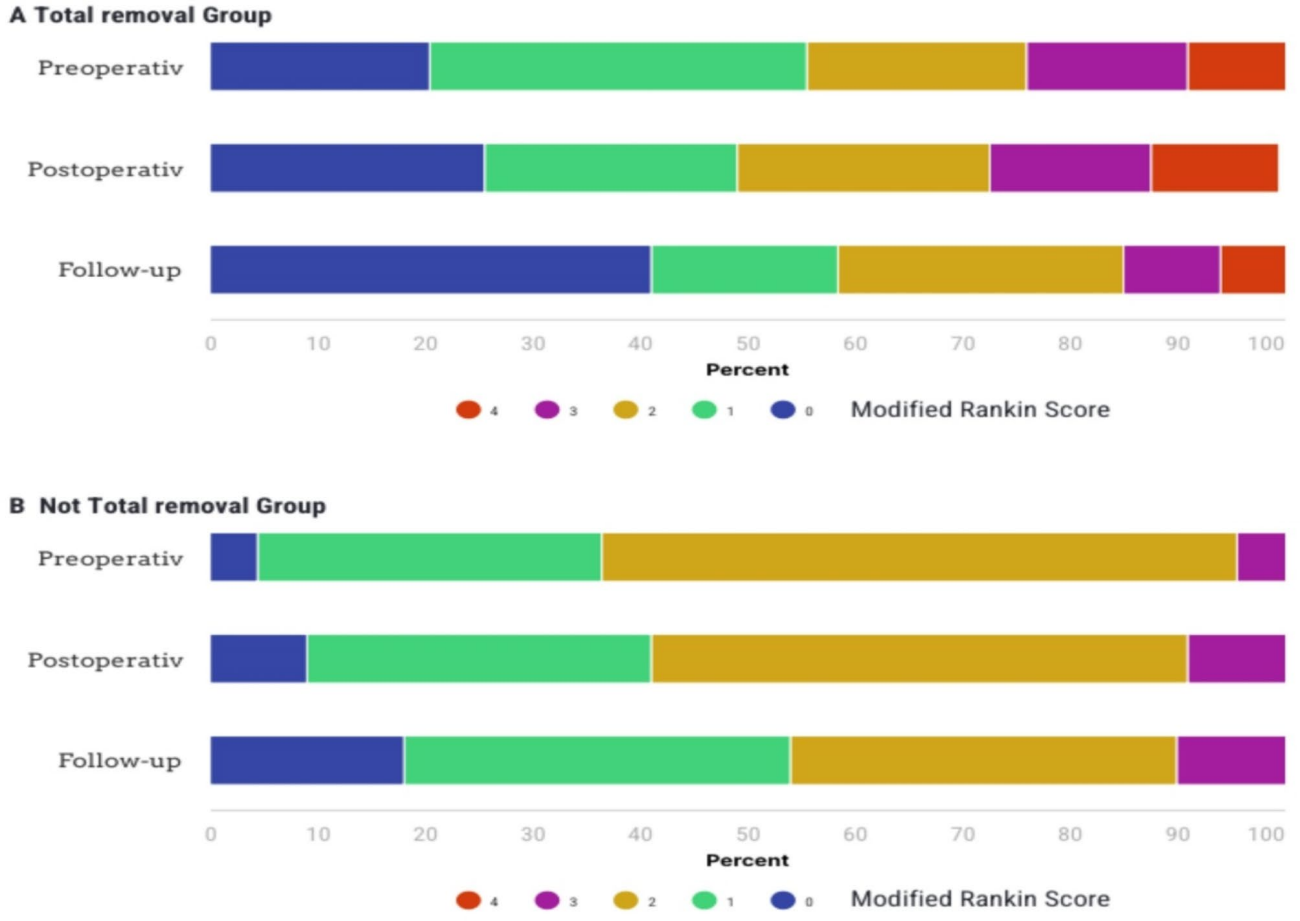
Modified Rankin Score comparing Total vs. Not total removal groups; immediate postoperative and in follow-up after 1 year.

In Fig. 3, the Modified Rankin Score (MRS) exhibited significant changes across both groups. For the total removal group, a substantial proportion of patients presented with excellent preoperative scores (0 or 1). However, postoperatively, there was a discernible decline, marked by a higher proportion of patients with scores ranging from 2 to 4. Nevertheless, at the follow-up (FU), there was a notable improvement, with a higher proportion of patients achieving scores of 0 to 1 compared to preoperative levels. Conversely, the proportion of patients with scores of 3 to 4 decreased postoperatively, Overall, there was improvement, though some patients stayed at score 2.

In the not-total removal group, in comparison to the total removal group, there were notably fewer patients with excellent preoperative scores (0–1). Postoperatively, there was a mixed response observed, with some patients experiencing deterioration (more score 3) while others showed improvement (more score 0). At the FU, the proportion of patients with excellent scores (0–1) significantly increased compared to the preoperative phase, suggesting substantial improvement. However, patients with deteriorated scores (3) postoperatively continued to exhibit deterioration at the FU, demonstrating a persistent decline compared to pre-/postoperative levels.

### Oncological outcome and predictors of regrowth

**Fig. 4:**
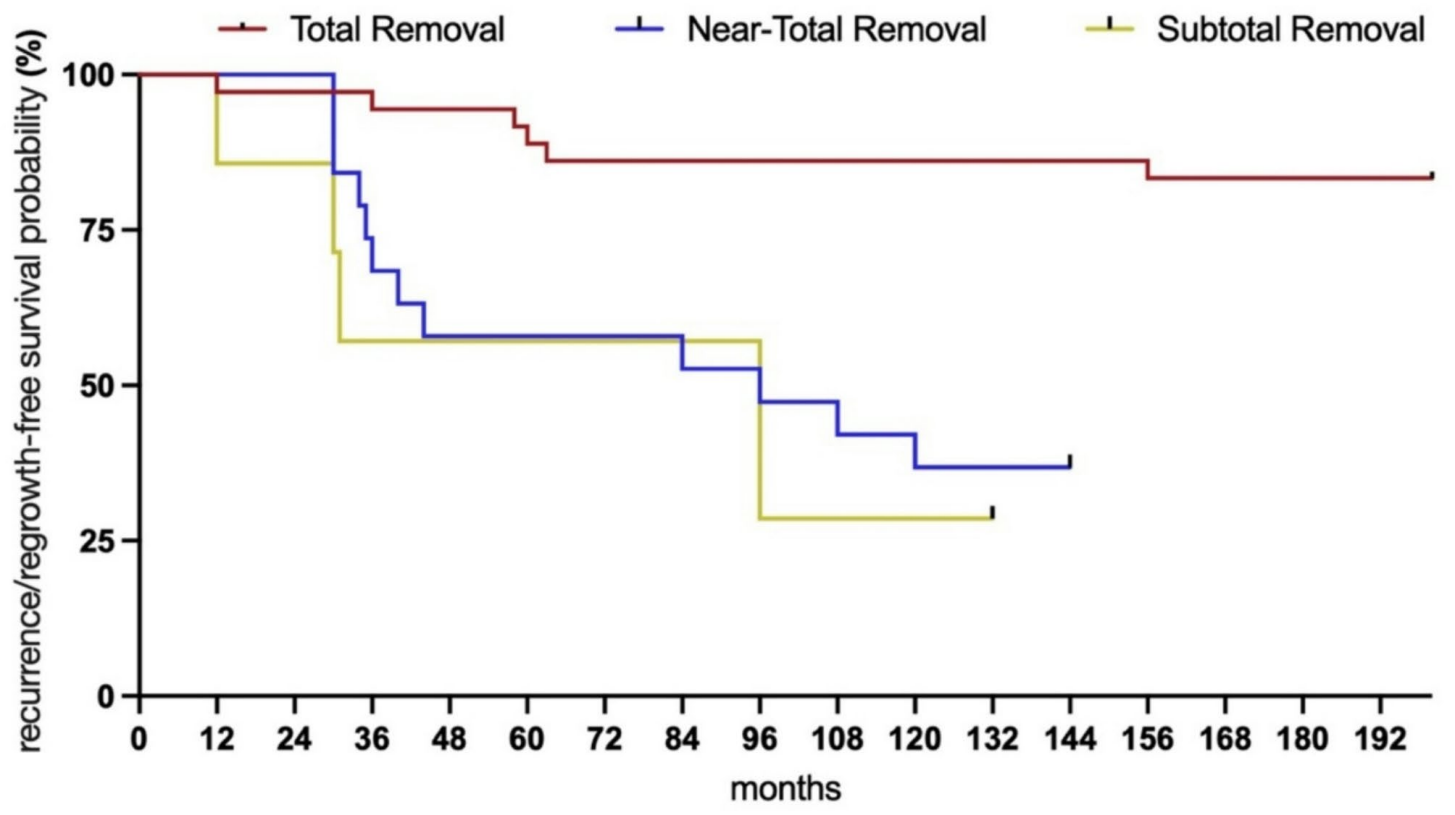
Kaplan–Meier recurrence/regrowth-free survival curve.

The Kaplan-Meier curves (Fig. 4) illustrate the survival probabilities for each group. In this context, ‘survival probabilities’ refer to the likelihood of remaining free from recurrence or regrowth of the epidermoid lesions as observed in follow-up MRIs. An event was defined as follows: (A) After total removal, it refers to the appearance of a new diffusion-restricted area on MRI, indicating epidermoid recurrence during follow-up. (B) After near-total or subtotal removal, it indicates the growth progression of remnants of restricted diffusion on MRI, indicating epidermoid regrowth. As shown in Fig. 4, Near-total removal group starts with above 90% survival probability, which declines to 35% over the observation period, with a median survival of 84 months. The “Subtotal” group shows a similar initial survival probability, but a steeper decline, leading to a 25% recurrence-free rate and a median survival of 96 months. The “Total removal” group exhibits the best outcomes, starting above 95% and maintaining a high probability of survival, with 75% remaining recurrence-free at the follow-up’s end.

Concerning the predictors of regrowth, CPA lesions with supratentorial extension or those crossing the midline in the prepontine area showed an odds ratio (OR) of 2.67 (95% CI: 0.33–21.73, *p* = 0.359) (Table 4). Lesions with less adhesion were significantly associated with a lower likelihood of regrowth (OR: 0.0625, 95% CI: 0.0083–0.4713, *p* = 0.007). In contrast, highly adhesive tumors demonstrated a significantly increased risk of regrowth (OR: 16.00, 95% CI: 2.12–120.65, *p* = 0.007).

**Table 4:** Impact of Lesion Extension (Midline/Supratentorial) and adhesion severity on epidermoid regrowth.

Parameter	Odds Ratio (OR)	95% Confidence Interval (CI)	*P*-Value
No Extension (Reference)	**-**	**-**	**-**
CPA lesions with supratentorial extension or those crossing the midline in the prepontine area	2.67	0.33–21.73	0.359
Less Adhesive Tumor	0.0625	0.0083–0.4713	0.007
More Adhesive Tumor	16.00	2.12–120.65	0.007

### Complications

The overall incidence of postoperative surgical complications was 21.5%. Bleeding occurred in 3 cases (4.5%), with subdural bleeding in 2 cases necessitating further surgery. Infections were noted in 4 cases (6%), comprising one wound infection, treated operatively, and three cases of meningitis, treated conservatively. Hydrocephalus was diagnosed in 3 patients (4.5%), with one preoperative case, managed via VP-Shunt implantation. CSF fistula occurred in 4 patients (6%), mainly subcutaneous; three were treated with a lumbar drain and one surgically (Table 5). Non-surgical complications were observed in 4 cases (6%), including pneumonia in 3 patients (4%) and sinus venous thrombosis in 1 patient (2%).

**Table 5:** Postoperative surgical complications.

Type of Complication					Overall
Bleeding	Intracerebral	Operation	Conservative		3 (4.5%)
			1		
	Subdural	2			
	Epidural				
Infection		Operation	Conservative		4 (6%)
	Wound Infection	1			
	Meningitis		3		
	Abscess				
Hydrocephalus		Lumbar Drainage	EVD	VP-Shunt	3 (4.5%)
	Pre/postoperative persistent			1	
	New postoperative			2	
CSF Fistula		Lumbar Drain	Operation		4 (6%)
	Subcutaneous	3	1		
	Wound healing disorder				
**Overall**					14/65 (21.5%)

Table 6 presents a comparison of data from different studies on epidermoid lesions, examining various factors, including the extent of resection (EOR), neurological functional outcomes, endoscopic-assisted operations, and recurrence rates.

**Table 6:** Recent studies on intracranial epidermoids since 2000 (*n* > 25).

Authors & years	No. of cases	Location (No of. cases)	Methodology	Follow-up period (Mean)	EOR (% of Patients)	Neurological functional Outcome (direct postop)	Neurological functional Outcome in follow-up (compared to direct postop)	Endoscopic assistance operations (%)	Recurrence rate
Kobata et al., 2002^24^	30	CPA (30)	Retrospective Analysis	138 mos	TR (57%)	-ND	-ND	-ND	Crude recurrence free (90%)*
Akar et al., 2003^42^	28	CPA (17), 4th V/FM (3), sylvian fissure (4), occipital lobe (2), lateral ventricle (1), & intradiploic area (1)	Retrospective Analysis	72 mos	Total/radical subtotal (75%), subtotal (25%)	-ND -New CN deficits: 46.4%	-ND	-ND	Crude recurrence free (93%)*
Gopalakrishnan et al., 2014^28^	50	CPA (37), 4th Ventricle (13)	Retrospective Analysis	111 mos	TR 62%, STR 38%	-ND	-ND	-ND	5-yr recurrence free: -TR: 91% -STR: 7%
Lynch et al., 2014^43^	29	CPA (13) & parasellar (6), cerebellar (5), temporal (5), & frontal (4) areas	Retrospective Analysis	86 mos‡	GTR (73%) & STR (27%)‡	-ND	-ND	-ND	-ND
Aboud et al., 2015^18^	38	Primary lesions: CPA (18), prepontine cistern (4), suprasellar (2), & MF (2) Recurrent lesions: CPA (6), suprasellar region (3), prepontine cistern (2), & parasellar region (1)	Retrospective Analysis	56.8 mos	GTR (73% of those w/ primary lesion & 17% w/ recurrence)	-ND -New CN deficits: 45.6%, in recurrence group: 58%	-ND	-Overall: 28.2%	Crude recurrence-free (85% of those w/ primary tumor & 58% w/ recurrence) †
Rehman et al., 2018^44^	38	CPA (18), (frontal lobe [4], temporal lobe [9], & temporal & parietal lobes w/ CS [1]), & suprasellar region (6)	Retrospective Analysis	-ND	-ND	-ND	-ND	-ND	-ND
Vernon et al. 2020^20^	139	CPA (139)	Retrospective Analysis	27 mos	TR 67%, NTR 3%, PR 2%	-ND -New CN deficits: 41%	-ND -Development of the new CN deficits: Better: 78% Stable: 20% Worse: 2%	-Overall: 40%	2.5-yr recurrence free: 85%
Hasegawa et al. 2022^8^	63	Posterior fossa (33), lobar/interhemispheric location (11), combined supratentorial & infratentorial area (11), Middle Fossa 3 & pineal region (5)	Retrospective Analysis	88.7 mos	GTR 20%, NTR 24% STR 35% & PR 22%	-ND -New CN deficits: 31%	-ND	-ND	5-yr recurrence free (66%)
Present Study	66	CPA and prepontine (49), Supratentorial (9). 4th Ventricle (7)	Retrospective Analysis	82.02 mos	TR 61%, NTR 27.6% STR 10.7%	-Better: 11.9% -Stable: 50% -Worse: 10.3% - New CN deficits: 27.7%	-Better: 36.4% -Stable: 51.1% -Worse: 12.4%	-Overall: 33.8% -CPA and prepontine region:36.7%, Supratentorial: 33.3%, 4th Ventricle: 14.2%	5-yr recurrence free: TR: 95% NTR: 45% STR 30%

ND = no data. GTR = gross total resection. NTR = near total resection. STR = subtotal resection. PR = partial resection. TR = total resection. CN = cranial nerves. * A detailed definition of recurrence was not provided. † Recurrence was defined as progressive symptoms with lesion enlargement. ‡ Data included patients with epidermoid and dermoid lesions.

## Discussion

This 20-year retrospective study provides substantial evidence that, despite the challenges presented by the proximity of intracranial epidermoid lesions to critical neurovascular structures, total removal is linked to improved long-term symptom management and reduced recurrence rates. Notably, this approach does not substantially elevate the risk of short-term neurological deficits or complications, making it a preferred strategy in the surgical management of these lesions.

Despite their benign and non-malignant histology, the management of intracranial epidermoid lesions presents a significant challenge for neurosurgeons^16–18^. This complexity largely stems from the tendency of these lesions to adhere to critical neurovascular structures, cranial nerves, and the brainstem^9^. While there is a prevailing tendency in the literature to favor total resection, significant concerns persist regarding the prevalence of postoperative neurological deficits^5,18–20^. In this study, we aimed to elucidate the intricate balance between maximizing the extent of resection and preserving neurological function in the management of intracranial epidermoid lesions. We found that radical resection enhances long-term symptom improvement without increasing short-term neurological deficits or complications ratio compared to total resection. Additionally, total cyst removal consistently led to better clinical outcomes in long-term follow-ups, suggesting its superiority in reducing recurrence and improving overall prognosis.

In our series, the peak incidence of intracranial epidermoid lesions was observed between the third and fourth decades of life, with a tendency to a higher prevalence in males. This trend aligns with findings reported in other case series in the literature^9,18,21^. The predominant clinical manifestations in our series were trigeminal nerve involvement and dizziness, followed by hearing difficulties or loss, headaches, and gait disturbances. These symptoms are indicative of compression of epidermoid tissue on adjacent structures and vary according to the lesion’s location. Trigeminal nerve involvement, the most frequent symptom observed, correlates with the high prevalence of location in the Cerebellopontine Angle (CPA) and prepontine cistern in our series, accounting for 60.4%, compared to the 38.9–50% range reported in existing literature^19,20,22,23^. The CPA location typically leads to direct nerve compression at the root entry zone or its displacement and compression against blood vessels, resulting in the symptoms we observed. This pattern of presentation is more common with epidermoid lesions in the CPA, as compared to other CPA lesions such as acoustic neurinomas or meningiomas^20,24,25^.

In our study, the surgical approach was consistently aimed mainly at achieving total removal, considering three important factors: the patient’s age, degree of involvement of neurovascular structures and the patient’s neurological status before the operation^14^. Total removal was achievable in over 50% of cases across all locations, with the success rate peaking at 66.6% in supratentorial lesions, which is comparable with other series in the literature^5,18,26^. The rationale for striving for total removal is multifaceted. Primarily, total removal is pursued to reduce the risk of recurrence. It’s suggested that the growth rate for recurring epidermoid correlates with the patient’s age at initial presentation, plus approximately nine months, and can sometimes be faster than those expectations^21^. Al-Mefty and colleagues have highlighted the challenges and increased neurological complications associated with repeated surgeries on these lesions. Each subsequent surgery not only accumulates deficits and morbidity but also reduces the likelihood of achieving total removal due to severe adhesions and inflammatory reactions^18^. In our study, the first sign of recurrence on imaging after total removal was observed only in 25% of the patients compared to 64-75% progression on imaging after non-total removal in a mean follow-up period of 82 months. Hasegawa et al. reported in their series that total/near-total removal led to a 10-year recurrence-free survival rate of 61% and a 10-year intervention-free survival rate of 100%^26^. Our research findings indicated that, after total removal 75% remained recurrence-free at the follow-up’s end. The reason for recurrence over the long-term follow-up in patients who underwent totalremoval remains a subject of speculation. One hypothesis is that the spatial resolution of diffusion-weighted imaging might not be adequate to completely rule out the presence of minuscule remnants, or that capsule remnants without diffusion restriction may remain that are not visible on imaging^26^. However, the decision on the optimal timing for follow-up surgery in cases of recurrence remains a topic of debate among neurosurgeons. The dilemma lies in choosing between early intervention at the initial sign of radiological recurrence and delaying surgery until symptoms reappear. Given the uncertainties associated with radiological interpretations, the prevailing approach tends to favor waiting for substantial radiological proof of recurrence or the reemergence of clinical symptoms before proceeding with a second surgery^16,27–30^. Additionally, it aims to minimize complications such as aseptic meningitis and hydrocephalus, which are more prevalent with leaving epidermoid. These complications likely arise from the residual lesion’s ongoing contact with brain tissue and cerebrospinal fluid^16–18,23,27^, or cyst content spillage during not-total removal^9^. Postoperative communicating hydrocephalus is thought to result from impaired CSF absorption due to chemical meningitis^9^. Aseptic meningitis cases, typically transient and self-limiting, are effectively managed with steroids^9,18^. In our series, it may be hypothesized that the high rate of total resection correspondingly resulted in a notably low incidence of postoperative hydrocephalus and aseptic meningitis, 4.5% and 5%, respectively.

The surgical approaches to remove the epidermoid lesions were determined based on their location and extent to provide adequate exposure. Approximately 80% of epidermoids located in the CPA and prepontine cistern were resected using retrosigmoid craniotomy. This technique provides good visualization while minimizing the need for extensive cerebellar retraction^20^. For supratentorial lesions, pterional and subtemporal craniotomies were employed. In cases of epidermoid lesions situated in the fourth ventricle, a median suboccipital craniotomy was performed. A two-stage operation through 2 different approaches, aimed at the total removal of giant epidermoids extending from the infratentorial to the supratentorial regions, as described by other authors in the literature^7,31^, were used as well.

Endoscopic-assisted removal was employed in 34% of the cases based on the surgeon’s discretion and was primarily used in challenging operations. Total removal was achieved in 54% of endoscopic-assisted cases. While the endoscope aided in lesion removal, we think that without its assistance, these complex cases might have resulted in less favorable outcomes. This is also consistent with the literature, as Tuchman et al. reported that in their case series, total or near-total removal was achieved in 54% of endoscopically assisted cases, with a 31% improvement in cranial nerve function^32^. Employing an endoscope for both inspection and excision of even small membrane remnants can reduce the need for extensive cerebellar retraction and cranial nerve manipulation, potentially minimizing surgical trauma and enhancing patient outcomes^33^. The endoscope provides a wider field of view, superior illumination, and the ability to visualize around corners, which is particularly advantageous in the narrow and angled corridors of the cerebellopontine angle, where the microscope’s straight-line visualization is limited^34^. This capability helps ensure a more thorough resection and reduces the risk of leaving behind hidden remnants, which could contribute to recurrence.

Furthermore, endoscopic-assisted techniques have proven valuable in confirming complete lesion removal, offering improved intraoperative inspection by accessing areas not directly visible under the microscope^32^. By bringing the light source closer to the operative field, the endoscope allows for high magnification with increased depth of focus, aiding in the meticulous dissection of lesion remnants while preserving critical neurovascular structures^35^. However, it is important to acknowledge the limitations of endoscopic techniques, including the lack of stereoscopic depth perception, restricts visualization beyond the tip, and the potential for obscured vision in the event of bleeding, which could complicate surgical maneuvers^36^. Hence, as highlighted by Tatagiba and colleagues, ensuring adequate craniotomy exposure remains crucial for optimal visualization, safe manipulation and dissection, control over the surgical area, and consistent irrigation, despite the advantages offered by endoscopic use^14^.

Over the past three decades, the introduction of microsurgical techniques has dramatically reduced morbidity and mortality associated with intracranial epidermoid lesions^5,19^. Consequently, the primary concern for neurosurgeons has shifted towards managing cranial nerve deficits that arise during the treatment of these conditions and improvement of clinical outcome^18^. In our study, we observed that the rate of immediate postoperative symptom improvement was comparable in cases of total and not-total lesion resection, with 12.6% in the total resection group versus 10.5% in the not-total removal group. Similarly, the incidence of new postoperative symptoms was relatively similar between these groups, being 25.3% for total removal and 32% for not-total removal. Nonetheless, it was noted that most of these postoperative deficits typically show resolution or significant improvement as time progresses. Consistently, longitudinal data over a year revealed, in our series, a more pronounced improvement in symptoms among patients who underwent total removal (41.7%) compared to those with subtotal removal (28%). Various factors may contribute to reducing the frequency of new neurological deficits post-surgery. These include the implementation of microsurgical methods complemented by endoscopic support and the proficiency of the surgical team. Furthermore, to minimize the risk of vascular damage, several steps can be taken, including a thorough review of preoperative imaging, such as 3D-CISS and TOF-angiography, to accurately identify vascular structures. Intraoperative hemodynamic control measures, such as maintaining optimal mean arterial pressure (MAP), using warm irrigation solutions, and, when necessary, administering vasodilatory agents like Nimodipine, can further aid in vascular preservation^37–39^. Additionally, intraoperative neurophysiological monitoring, as well as careful consideration of leaving a thin layer of membrane on critical vascular structures when adhesion is significant, may help protect vascular integrity during surgery. Additionally, the marked improvement in neurological symptoms observed during long-term follow-up in the total removal group can be attributed partly to the effective dissection plane established between the capsule and the neurovascular and cranial nerves structures, enhancing the recovery of those structures^28^.

This observation was also evident in the modified Rankin Score, which indicated an improvement in long-term postoperative outcomes compared to preoperative scores in both groups. Notably, this trend was more pronounced in the total removal group. A similar trend in the enhanced improvement of the Modified Ranking Score in the total resection group has been reported in the studies conducted by Schiefer and Link, as well as by Gopalakrishnan et al., following the resection of CPA Epidermoid lesions^28,40^.

Our study contributes to the literature by demonstrating that total removal of epidermoid lesions is not only associated with significantly lower regrowth rates but also with better long-term neurological recovery. Additionally, we provide a detailed analysis of the role of endoscopic assistance in lesions removal and identify adhesion severity and lesions extension as key predictors of recurrence. These findings offer a more refined approach to risk assessment, aiding in surgical planning and long-term follow-up strategies.

Furthermore, A distinguishing strength of our study, which sets it apart from others, lies in our methodology for classifying surgical outcomes as total or not-total resection. Unlike other research that often relies on the subjective impressions or descriptions provided by surgeons in operative reports, our classification was based on objective imaging analysis; The MRIs were independently analyzed by two experienced neurosurgeons. In cases where the classification of the MRIs differed between them, a blinded senior neurosurgeon was consulted to further enhance the reliability and accuracy of the findings.

This study, while providing valuable insights into the management of intracranial epidermoid lesions, is subject to several limitations. The retrospective nature of the analysis, based on a sample of 55 patients from a single institution, may introduce biases such as selection and recall bias, potentially affecting the generalizability of the findings. Some patient records may be incomplete or missing critical data points, which could influence the analysis and conclusions. Observer biases including Data interpretation, particularly regarding imaging findings and surgical reports, may be influenced by the subjective judgment of surgeons and analyst. Here, Adhesion severity was subjectively assessed based on surgical reports and imaging, which could introduce observer bias and variability in defining the extent of adhesion. Information bias like variability in data recording methods, such as differences in surgical documentation styles or postoperative imaging protocols over the years, may introduce inconsistencies in the dataset. Furthermore, the subjective nature of surgical decision-making and variability in surgical approaches could influence outcomes. The outcomes of surgical management may vary depending on the experience and skill level of the operating surgeon, which could introduce variability in results and affect the reproducibility of the findings in other centres. Selection Bias in Endoscopic-Assisted Cases, where the decision to use endoscopic assistance was made at the discretion of the surgeon, which may introduce selection bias and limit the generalizability of its reported benefits. The reliance primarily on DWI sequences for postoperative follow-up, with their inherent limitations in detecting microscopic remnants, may have led to underestimation of residual disease or recurrence; DWI sequences often have thicker slices compared to other MRI sequences because this helps improve image quality by reducing noise and motion-related distortions^41^. However, thicker slices can make it harder to detect small remnants, as they might blend with surrounding tissues, leading to less precise imaging. Despite the thicker slices, DWI provides a high specificity for detecting intracranial epidermoid tissue, which is why we used it as the basis for classifying the extent of removal. The long-term follow-up of patients was not uniform, and the potential underestimation of recurrence rates due to the resolution limits of postoperative imaging, especially in the early beginning of the series, must be considered. Given that recurrences of epidermoid lesions can potentially occur several decades post-surgery, an extended observation period is mandatory.

## Conclusion

In conclusion, this 20-year retrospective study offers significant insights into the long-term management of intracranial epidermoid lesions. Despite the inherent challenges posed by their proximity to critical neurovascular structures, the approach of total removal was linked to improved long-term symptom management and reduced recurrence rates in our series, without substantially elevating short-term neurological deficits or complications. In light of our findings, we recommend the total removal of epidermoid lesions as the optimal approach in their surgical management, while simultaneously emphasizing that the preservation of functional integrity must remain a top priority throughout the surgical process. Furthermore, total removal also diminishes the likelihood of subsequent surgeries and their associated complications, thereby offering a more favorable long-term prognosis for patients.

## Data Availability

The datasets analyzed during the current study are available from the corresponding authoron reasonable request.
